# Clostridioides difficile Infection in Healthy Children After Antibiotic Use: A Report of Two Cases

**DOI:** 10.7759/cureus.90928

**Published:** 2025-08-25

**Authors:** Kaho Shimizu, Ryuichi Nakagawa, Mari Okada, Saori Amano, Susumu Hosokawa

**Affiliations:** 1 Department of Pediatrics, Musashino Red Cross Hospital, Tokyo, JPN

**Keywords:** antibiotics, clostridioides difficile, clostridioides difficile infection, diarrhea, gastrointestinal hemorrhage, pediatric

## Abstract

Although* Clostridioides difficile* infection (CDI) commonly affects the elderly, hospitalized, or immunocompromised patients with underlying diseases, cases of community-acquired CDIs in previously healthy children have recently been reported. Here, we describe the cases of two healthy children who developed CDI after the use of antibiotics. Patients were administered either high-risk antibiotics for CDI or a combination of multiple low-risk antibiotics. The clinical characteristics of the patients that differentiated CDI from antibiotic-associated diarrhea (AAD) include bloody stools, fever, and an elevated inflammatory response. Even in healthy children with a history of low-risk antibiotic use, CDI should be considered in the differential diagnosis of prolonged diarrhea or bloody stools, highlighting the importance of a thorough antibiotic exposure history.

## Introduction

*Clostridioides difficile* is a gram-positive spore-forming anaerobic bacterium. It is a leading cause of antibiotic-associated diarrhea (AAD) and pseudomembranous colitis (PMC). It produces spores that allow it to survive in severe environments such as acidic, alkaline, high-temperature, and aerobic conditions. These characteristics facilitate the presence of *C. difficile* in the intestines of both animals and humans. The key pathogenic factors include spore production, high motility, capsule formation, hydrolytic enzymes, adherence factors, and toxins. The primary pathogenic mechanisms of *C. difficile* are derived from two major toxins: A and B. These toxins cause intestinal inflammation, resulting in the characteristic gastrointestinal symptoms of diarrhea and colitis [1]. Some hypervirulent strains, such as the BI/NAP1/027 strain, have emerged since 2002 and are considered significant threats by the Centers for Disease Control and Prevention [2].

The epidemiology of *C. difficile* infection (CDI) varies across age groups. The reported intestinal carriage rate among Japanese adults is approximately 7.6% [3]. Although CDI is widely recognized in adults, its prevalence and presentation differ among children. Healthy neonates and infants often carry *C. difficile* without developing symptoms, indicating natural colonization or immunization against toxins [4].

Common risk factors for CDI include advanced age, hospitalization, and underlying comorbidities [5-8]. Despite the absence of these risk factors, cases of CDI in otherwise healthy children are being increasingly recognized [9]. This underscores the importance of considering CDI in the differential diagnosis of pediatric enteritis, even in the absence of common risk factors. In the present case report, we aim to elucidate the clinical factors related to pediatric CDI and highlight the importance of including this disease in the differential diagnosis of pediatric enteritis by describing two cases of CDI in previously healthy patients.

## Case presentation

Case one involved a nine-year-old boy who was referred to our institute because of bloody stools. His medical history included three episodes of febrile urinary tract infections at seven months, one year, and eight years of age. However, the patient did not require admission for more than a year prior to the current presentation. Prophylactic cefaclor was administered since infancy to prevent recurrent febrile urinary tract infections. Long-term low-dose clarithromycin therapy was initiated 14 days before the referral visit for sinusitis. Six days later, the patient developed fever, abdominal pain, vomiting, and diarrhea, which led to the initiation of fosfomycin treatment. His symptoms did not resolve completely, and bloody stools appeared on the day of referral. He had no dietary history of meat consumption associated with CD.

The patient’s body temperature was slightly elevated at 37.6°C; however, other vital signs were stable. He presented with tenderness in the left abdomen. Laboratory data revealed no significant abnormalities except for a slightly elevated C-reactive protein (CRP) level (Table 1). Stool samples tested positive for CD antigens and toxins; however, no other specific pathogenic bacteria were identified. The patient was diagnosed with CDI, and treatment with metronidazole was initiated. Following treatment, his symptoms resolved, and he was discharged within six days.

**Table 1:** Laboratory data of the present cases CRP: C-reactive protein; *C. difficile*: *Clostridioides difficile*

Parameters	Case one	Case two	Reference range (95% CI)
WBC (/μL)	9200	14000	4500 - 13500
Hemoglobin (g/dL)	13.5	15.1	12 - 14
Hematocrit (%)	39.8	42.6	37.5 - 42.5
Platelet (×10^4^/μL)	50.9	40.3	15 - 40
Albumin (g/dL)	3.7	4.2	4.0 - 5.0
Blood urea nitrogen (mmol/L)	3.4	14.3	2.1 - 7.1
Creatinine (μmol/L)	27.4	36.2	35.4 - 70.7
Sodium (mmol/L)	138	136	138 - 145
Potassium (mmol/L)	4.4	4.0	3.4 - 4.7
Total bilirubin (μmol/L)	6.8	8.6	1.7 - 17.1
Aspartate aminotransferase (IU/L)	19	20	17 - 39
Alanine aminotransferase (IU/L)	12	17	4 - 23
Creatine kinase (IU/L)	46	58	52 - 305
CRP (μg/L)	19600	19200	1000 - 10000
*C. difficile *antigen	Positive	Positive	Negative
*C. difficile *toxin	Positive	Positive	Negative

Case two involved a 10-year-old boy referred to our institute with prolonged abdominal pain and diarrhea. His father had ulcerative colitis, but the boy had no significant medication use or past medical history. He had been experiencing fever, abdominal pain, and diarrhea, which led to the initiation of cefditoren pivoxil administration 18 days prior to referral. His fever and gastrointestinal symptoms initially improved, although abdominal pain and diarrhea recurred seven days before referral. There was no history of consuming meat products that could cause CD.

On presentation, his vital signs, including body temperature, were within the normal range. He exhibited left abdominal tenderness. Laboratory data revealed slightly elevated white blood cell counts and CRP levels (Table 1). Stool samples tested positive for CD antigen and toxin; therefore, the patient was diagnosed with CDI. No other specific pathogenic bacteria were identified. Metronidazole treatment was initiated, and the gastrointestinal symptoms resolved. The patient was discharged within four days.

## Discussion

This report presents the development of CDI in previously healthy children without underlying conditions, characterized by prior antibiotic use. *Clostridioides difficile* infection is defined by the presence of gastrointestinal symptoms, such as diarrhea, in conjunction with either a stool test positive for *C. difficile *toxins or antigens, or endoscopic or histopathological evidence of pseudomembrane [10]. According to the Infectious Diseases Society of America guidelines, CDI in adults is classified as non-severe, severe, or fulminant CDI based on a combination of white blood cell count, serum creatinine level, and clinical features [10]. Severity of CDI in children is poorly defined [9], whereas our cases were classified as non-severe based on clinical findings (Table 1). The initial management in CDI involves discontinuing any non-CDI antibiotics to prevent further intestinal microbial disruption [9]. The choice of antibiotics for CDI treatment depends on the number and severity of episodes. For an initial or first recurrence of non-severe CDI, oral metronidazole or oral vancomycin for 10 days is recommended. For an initial episode of severe or fulminant CDI, or for a second or greater episode of recurrent CDI, oral vancomycin is recommended over metronidazole [9]. Our cases were successfully treated with metronidazole. Additionally, our cases were not associated with any severe CDI complications in children, such as pseudomembranous colitis, pneumatosis intestinalis, toxic megacolon, perforation, peritonitis, and shock with multisystem failure.

Unlike adults, a significant proportion of CDI cases in children are community-acquired. Notably, previous reports indicated that approximately 75% of CDI cases in children are community-associated rather than healthcare-associated, and that 13% of community-acquired CDI cases occurred in patients under 18 years of age [9,11]. This tendency indicates the importance of considering CDI in younger non-hospitalized pediatric populations. Although CDI is often considered in hospitalized patients or those with recent healthcare exposure, the increasing incidence of community-acquired CDI indicates that a lack of an inpatient history should not preclude the possibility of CDI in children [11,12].

The development of CDI is closely linked to several risk factors. These include advanced age, hospitalization, underlying comorbidities (neoplastic disease, immunodeficiency, solid organ transplantation, or enteral feeding), and antibiotic exposure [5-8]. Antibiotics disrupt normal gut microbiota, creating an ecological niche that allows CD to proliferate and produce toxins [7]. In our study, antibiotic administration was a significant risk factor. Different antibiotic classes carry varying risks of CDI development. High-risk antibiotics include clindamycin, fluoroquinolones, and third- and fourth-generation cephalosporins [7,8]. One of our patients received cefditoren pivoxil, which belongs to the high-risk category (third-generation cephalosporins). In another case, although only low-risk antibiotics were used, multiple agents were administered. Previous reports suggested that the use of multiple antibiotics, even if considered low-risk, can synergistically increase the risk of CDI [13]. Previous Japanese reports on the clinical features of other cases (Table 2) also indicated that high-risk antibiotic exposure and the combined use of agents were associated with a risk of CDI development. This highlights the importance of a comprehensive review of antibiotic exposure, not focusing solely on high-risk single agents.

**Table 2:** Comparison between previously reported cases in Japan and our two cases of pediatric CDI in patients without underlying conditions CDI: *Clostridioides difficile* infection; WBC: White blood cell; CRP: C-reactive protein; AMPC: Amoxicillin; CAM: Clarithromycin; FRPM: Faropenem; FOM: Fosfomycin; AMPC/CVA: Amoxicillin/clavulanic acid; CDTR-PI: Cefditoren pivoxil; ABPC: Ampicillin; CLDM: Clindamycin; AZM: Azithromycin; CTRX: Ceftriaxone; CCL: Cefaclor; VCM: Vancomycin; MNZ: Metronidazole

Characteristics	Fujii et al. [16]	Mimura et al. [17]	Mimura et al. [17]	Kikuno et al. [18]	Kimura et al. [19]	Present case one	Present case two
Age (in years)	5	4	3	15	7	9	10
Gender	Male	Male	Female	Male	Female	Male	Male
Chief complaints	Abdominal pain, diarrhea, bloody stool	Bloody stool	Bloody stool	Fever, diarrhea	Fever, diarrhea, abdominal pain	Fever, bloody stool	Abdominal pain, diarrhea
Interval from first antibiotic exposure to admission	Two months	Two months	14 days	19 days	8 days	14 days	18 days
Antibiotics received before admission	AMPC, CAM, FRPM, FOM	AMPC/CVA, FOM	AMPC, CDTR-PI	ABPC, CAM	CAM, CLDM, AZM, CTRX, CDTR-PI	CAM, FOM, CCL	CDTR-PI
Body temperature (°C)	37.0	37.3	36.1	38.0	38.8	37.6	37.3
WBC (/μL)	8800	No data	No data	16300	8800	9200	14000
CRP (μg/L)	22000	No data	No data	1300	84000	19600	19200
Diagnostic method	*C. difficile *antigen, *C. difficile* toxin	*C. difficile *toxin	*C. difficile* toxin, colonoscopy	*C. difficile* antigen, colonoscopy	*C. difficile* toxin, colonoscopy	*C. difficile *antigen, *C. difficile* toxin	*C. difficile *antigen, *C. difficile* toxin
Treatment	Oral VCM	Stop antibiotics	Oral VCM	Oral MNZ	Oral VCM	Oral MNZ	Oral MNZ

It has been hypothesized that the risk of developing CDI persists even after a long period of antibiotic treatment. Meta-analyses revealed that the risk of developing CDI was 1.37 times (odds ratio: 1.37, 95% confidence interval (CI): 0.94-2.01) and 2.12 times (odds ratio: 2.12, 95% CI: 1.64-2.76) higher even within 12 weeks after antibiotic discontinuation compared to that in individuals not exposed to antibiotics [8,14]. Shirley et al. described how antibiotic exposure to amoxicillin-clavulanate, cephalosporins, or clindamycin within the preceding 12 weeks is a risk factor for CDI [9]. Consistent with Shirley et al.'s report, all but one case in Table 2 involved the use of amoxicillin/clavulanic acid (AMPC/CVA), cephalosporins, or clindamycin within 12 weeks. Thus, while we focused on Japanese patients for a detailed comparison, we consider these Japanese cases in Table 2 to represent a portion of a worldwide sample of the pediatric population with CDI. This prolonged risk period is a crucial aspect of patient history-taking. Even if a patient does not report recent antibiotic use prior to symptom onset, a detailed history of antibiotic exposure within the preceding three months is essential.

It is crucial to differentiate CDI from other forms of diarrhea, particularly AAD, for appropriate management. Consistent with previous reports, one of our patients presented with bloody stools (Table 2). Although AAD often involves osmotic diarrhea due to carbohydrate malabsorption following changes in the gut flora [15], the presence of blood in the stool can be a strong indicator of inflammatory bowel damage characteristic of CDI and should, therefore, prompt consideration of *C. difficile* as the causative agent. Furthermore, compared to the general signs of AAD, CDI is more frequently accompanied by systemic inflammatory signs, such as fever and an elevated inflammatory response [15]. In our patients, at least one of these systemic signs (fever, elevated white blood cell count, or increased CRP level) was observed, suggesting their potential utility as useful markers in the differential diagnosis of pediatric enteritis (Table 2). These clinical features, along with comprehensive history taking, can lead to appropriate diagnostic testing for *C. difficile*, which can help avoid unnecessary broad-spectrum antibiotic use and ensure effective treatment.

## Conclusions

Identifying the risk of developing CDI is essential, particularly with regard to prior antibiotic exposure, even in children without any underlying medical conditions. Clinicians must also pay attention to low-risk antibiotics, particularly when used in combination, even when a long period (a maximum of 12 weeks) has passed since the initial antibiotic exposure. Additionally, clinicians should consider CDI in patients presenting with diarrhea (particularly bloody diarrhea), fever, or an elevated inflammatory response after the use of antibiotics and should conduct appropriate diagnostic testing, such as C. difficile antigen or toxin tests, to diagnose CDI.
